# Synopsis of Acanthocerini (Hemiptera, Coreidae) from Argentina

**DOI:** 10.3897/zookeys.305.3727

**Published:** 2013-05-29

**Authors:** José Luis Pall, María del Carmen Coscarón

**Affiliations:** 1 División Entomología, Facultad de Ciencias Naturales y Museo, Universidad Nacional de La Palta, Paseo del Bosque, CP 1900, La Plata, Argentina; 2 Facultad de Ciencias Exactas y Naturales, Universidad Nacional de La Pampa, Argentina, Uruguay 151 L6300CLB, Santa Rosa, La Pampa, Argentina

**Keywords:** Acanthocerini, genera, Argentina, key, redescription, distribution

## Abstract

Eight genera and 13 species of the tribe Acanthocerini are recorded in Argentina, i.e., *Athaumastus haematicus* (Stål), *Athaumastus macer* Brailovsky, *Athaumastus subcarinatus* (Stål), *Athaumastus subterlineatus* Bergroth, *Beutelspacoris sanchezi* Brailovsky, *Beutelspacoris dilatata* Casini, *Camptischium clavipes* (Fabricius), *Crinocerus sanctus* (Fabricius), *Dersagrena flaviventris* (Berg), *Dersagrena lacerdae* (Signoret), *Dersagrena subfoveolata* (Berg), *Thlastocoris hernandezi* Brailovsky and *Zoreva dentipes* Fabricius. Redescriptions are given for *Athaumastus haematicus*, *Athaumastus subcarinatus* and *Dersagrena flaviventris* with photographs of male and female genitalia of *Dersagrena subfoveolata*. *Zoreva* recorded from Argentina the first time. New locality records are given for Buenos Aires, Chaco, Formosa, Misiones, and Tucumán.

## Introduction

The Coreidae, commonly called “leaf-footed, pod- or squash-bugs”, are heavy bodied insects usually strongly elongate or broadly elliptical. This family includes some of the largest living heteropterans, as well as species that are delicate or slender (Schuh & Slater 1995). The family contains about 267 genera and more than 1884 species worldwide (Henry 2009). The basic suprageneric classification was established by Stål (1867, 1870) and Schaefer (1964, 1965), who provided the most comprehensive treatment. Packauskas (1994) gave keys to the subfamilies and tribes of the New World Coreidae and a checklist of published keys to genera and species, and Packauskas (2010) cataloged the New World Coreidae and provided a comprehensive introduction to the literature.

The most comprehensive treatment of the Coreidae for Argentina are by Pennington (1920, 1921) and Kormilev (1954), but only Pennington (1921) dealt with the Acanthocerini.

Many coreids are of great economic importance. As mentioned by Mitchell (2000), grains legumes, rice, cassava, cucurbits, tomatoes, garden vegetables, and various fruit and nut trees are among the crops attacked by coreids worldwide. According to Mitchell (2000), the Acanthocerini includes species of minor economic importance, i.e., *Athaumastus haematicus* (Stål) that attacks potatoes, cotton, sunflower, oranges and eggplant, *Dersagrena flaviventris* (Berg) on cotton, and *Camptischium clavipes* (F.) on castor bean and guaco (Bosq 1937, 1940; Mitchell 2000).

Knowledge of the South American fauna is poor; this is especially true in regard to economically important taxa. Comprehensive keys for identification of the species in the region have not been published.

Most of Argentina lies in the Neotropical faunal region. The country covers an area of 2,791,810 km^2^ and is bordered by Uruguay, Brazil, Paraguay, Bolivia, and Chile. Approximately 75% of the country is occupied by arid and semiarid areas, but rainforests are also present in the northeast, i.e., the Yungas and Paranaense regions. Coscarón (submitted) recorded 125 species in 48 genera of Coreidae living from Argentina.

The goal of this paper is to provide an illustrated key to the genera of Acanthocerini (Coreidae) from Argentina (Fig. 38), a diagnosis and redescription of some of its species, geographical distribution, and a list of species for each genus.

### Materials and methods

All specimens from this study are deposited in the collections of the Museo de Ciencias Naturales de La Plata (MLP), La Plata, Buenos Aires, Argentina (http://www.fcnym.unlp.edu.ar/abamuse.html). Photographs were compared with material of the Naturhistoriska Riksmuseet in Stockholm, Sweden (http://www.nrm.se/2.1286b10fdbe80efba80001.html) and the American Museum of Natural History in New York (http://www.amnh.org/). For the geographical distribution we used the program DIVA-GIS 7.1.7 (http://www.diva-gis.org/) and the distribution of those specimens for which global positioning system data were available was used for the construction of maps. Photographs were produced using a Kodak Easy Share (12 megapixels) camera and a magnifying Wild M-Stereomicroscope. The diagnoses of genera are taken from Brailovsky (1987) and O’Shea (1980), except as noted. Coreoidea Species File (Version 1.1/4.1) (http://coreoidea.speciesfile.org/HomePage.aspx) was consulted. All measurement are in millimeters.

### Key to the genera of Acanthocerini for Argentina

**Table:** 

1a	Pronotum slightly declivent (Fig. 31)	2
1b	Pronotum not slightly declivent	*Thlastocoris* Mayr
2a	Posterior femora tuberculate ventrally and smooth dorsally (Fig. 29)	3
2b	Posterior femora tuberculate ventrally and dorsally (Fig. 20)	5
3a	First antennal segment much longer than third segment, humeral angles not sharp (Fig. 30)	4
3b	First antennal segment much shorter than third segment, humeral angles sharp but hardly produced laterally (Fig. 26)	6
4a	Humeral angles of pronotum pointed	*Zoreva* Amyot & Serville (Fig. 29)
4b	Humeral angles of pronotum rounded	*Beutelspacoris* Brailovsky
5a	Dorsal surface of pronotum markedly tuberculate	*Camptischium* Amyot and Serville (Fig. 16)
5b	Dorsal surface of pronotum punctate but not tuberculate	*Crinocerus* Burmeister (Fig. 18)
6a	Antennifers narrowly but distinctly separated	*Dersagrena* Kirkaldy (Figs 21, 26)
6b	Antennifers meeting mesially	*Athaumastus* Mayr (Figs 1, 6)

## Taxonomy

### Genus Athaumastus

Mayr

http://coreoidea.speciesfile.org/Common/basic/Taxa.aspx?TaxonNameID=759

http://species-id.net/wiki/Athaumastus

Figs 1, 6

Athaumastus Mayr 1865: 431. Type species: *Crinocerus lugens* Stål 1855: 184; monotypic.

#### Diagnosis.

(After O’Shea 1980) Body medium-sized, robust, postocular tubercles well developed; antennifers large, placed close together, projecting anteriorly of tylus, with well developed spine on lateral surface; pronotum not very steeply declivent, lateral margins slightly nodulose, humeral angles rounded-angulate, posthumeral, posterior margins relatively smooth; anterior femora with or without distal spines on ventral surface, intermediate and posterior femora armed at least with small apical spines, posterior femora markedly curved, incrassate, especially in male; posterior tibiae flattened, armed with teeth along ventral margin, with large tooth halfway along ventral margin in male.

**Figures 1–10.**
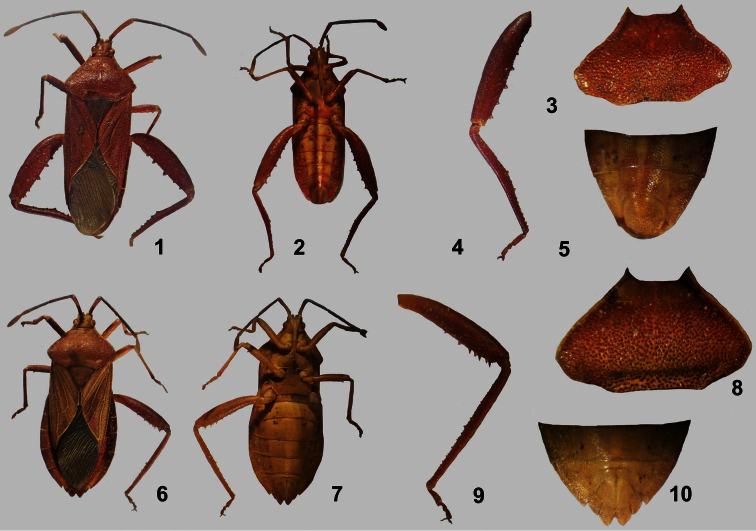
*Athaumastus haematicus* (Stål). **1–5** ♂, **1** dorsal view **2** ventral view **3** pronotum **4** hind leg **5** male genital capsule, ventral view **6–10** ♀ **6** dorsal view **7** ventral view **8** pronotum leg **9** hind leg **10** female genital segment, ventral view.

### Athaumastus haematicus

(Stål)

http://coreoidea.speciesfile.org/Common/basic/Taxa.aspx?TaxonNameID=779

http://species-id.net/wiki/Athaumastus_haematicus

Figs 1–5, 6–10 , 39

Crinocerus haematicus Stål 1859: 455.
Athaumastus haematicus Berg 1878: 85.–Pennington 1920: 13.–Pennington 1921: 36.–Bosq 1937: 113.–Hayward 1942: 72.–Hayward 1960: 30.–Quintanilla et al. 1968: 31.–Viana and Williner 1972: 27.–Quintanilla et al. 1976: 117.–Rizzo 1976: 34.–Viana and Williner 1978: 74.–Quintanilla et al. 1981: 147.

#### Redescription.

Male. n= 8. Total body length: 12.3-14.2: head length 1.0-1.2; head width 1.4-0.18; eye width 0.2-0.3; interocular space 0.8-0.9; preocellar distance 0.4; interocellar space 0.2. Rostrum: I 0.7-0.8, II 1.2, III 1.0, IV 0.64-0.7. Antennal segments: I 1.8-2.1, II 1.3-2.2, III 2.2-2.7 and IV 1.4-2.0. Pronotum length 2.3-2.9; width 4.6-6.4. Scutellum length 2.3, width 2.2. Length of abdomen with hemelytra: 8.6-9.8; length abdomen with hemelytra: 9.0-10.0; Abdomen width: 3.9-4.6. Dorsal coloration: Head brown tinged with red except antennal segments 2-3, bases of segments with brown tonalities. Pronotum brown, tinged with red. Scutellum dark read. Corium and clavus brown, tinged with red and hemelytral membrane dark brown, veins light brown. Connexival segments dark brown, tinged with red. Ventral coloration: Ground color light brown, tinged with red, mesosternun darker and abdomen not homogeneously dark pigmented. Legs dark brown, tinged with red. Structure: Pronotum rugose. Frontal angles rugose projecting with in as acute projecting spines, humeral angles with two rounded projections. Scutellum granulate. Metafemora with two rows of spines, tibia with small teeth basally. Hemelytra shorter than the abdomen.

Female. n=8. Total body length: 12.0-13.5; head length: 10.0; head width: 1.4-1.6; eye width: 0.2-0.3; interocular space: 0.8-0.9; preocellar distance: 0.4; interocellar space: 0.3. Rostrum: I 0.6-0.8, II 1.2, III 0.9, IV 0.7. Antennal segments: I 1.1-1.7, II 1.5-1.6, III 1.9-20.0 and IV 1.2-1.5. Pronotal length: 2.2-2.7; width: 4.2-5.0. Scutellar length: 1.7; width: 1.5. Length of abdomen with hemelytra: 9.0-11.0; length of abdomen without hemelytra: 9.6-11.3; abdomen width: 4.7-5.4. Dorsally and ventrally light brown. Anterior angles granulate; humeral angles with two rounded projections.

#### Specimens examined.

**Argentina**: Catamarca: 1♂ Andalgalá (27°36’02"S, 66°18’56"W), 1♂ Pomán (28°23’44.38"S, 66°13'06.91"W). Córdoba: 2♀ Cabaña (31°13'00.56"S, 64°22'00.32"W), 1♂ Makena (31°21'52.69"S, 64°10'18.54"W), 3♀ 2♂ Marull (30°59'43.72"S, 62°49'38.55"W), 1♂ Mar Chiquita (30°48'35.46"S, 62°52'31.68"W), 1♂ Rayo cortado (30°04'26.09"S, 63°49'25.99"W). 2♀ 3♂ Corrientes. Chaco: 1♀ Charata (27°13'06.31"S, 61°11'15.89"W), 1♀ El zapallar (26°32'17.85"S, 59°20'42.57"W), 8♀ 7♂ Gancedo (27°29'21.89"S, 61°40'31.52"W), 1♀ Resistencia (27°27'05"S, 58°59'10"W). Formosa: 2♂ Ingeniero Juárez (23°53'46.85"S, 61°51'37.88"W). 1♂ La Rioja. 1♀ San Luis. Santiago del Estero: 1♂ Río Salado (27°44'32.51"S, 64°21'16.97"W).

#### Distribution.

**Argentina**: Buenos Aires; Catamarca; Córdoba: Carlos Paz, El Sauce, San Javier; Corrientes: Alvear Department, Bella Vista Department, Berón de Astrada Department, Capital Department, Colón Department, Concepción Department, Concordia Department, Diamante Department, Empedrado Department, Esquina Department, General Paz Department, La Paz Department, Mburucuyá Department, Paraná Department, Paso de los Libres Department, Saladas Department, San Martín Department, San Miguel Department, San Roque Department, Santo Tomé Department, Victoria Department; Chaco; Entre Ríos: Concordia; Formosa; Jujuy; La Pampa; La Rioja; Mendoza; Misiones: Alem Department, Apóstoles Department, Cainguás Department, Concepción de la Sierra Department, Guaraní Department, Montecarlo Department, Oberá Department, San Javier Department, San Martín Department, 25 de Mayo Department; Neuquén; Salta; San Juan; San Luis: San Gerónimo, Suyuque; Santa Fé; Santiago del Estero; Tucumán.

### Athaumastus macer

Brailovsky

http://coreoidea.speciesfile.org/Common/basic/Taxa.aspx?TaxonNameID=781

Athaumastus macer Brailovsky 1993: 115.

#### Distribution.

**Argentina**: Tucumán

### Athaumastus subcarinatus

(Stål)

http://coreoidea.speciesfile.org/Common/basic/Taxa.aspx?TaxonNameID=783

http://species-id.net/wiki/Athaumastus_subcarinatus

Figs 11–14 , 40

Crinocerus subcarinatus Stål 1859: 455.
Athaumastus subfoveolatus Berg 1892: 66.–Coscarón 1998: 4.
Athaumastus subcarinatus Pennington 1920: 13.–Pennington 1921: 38.–Quintanilla et al. 1968: 31.–Quintanilla et al. 1976: 118.Viana and Williner 1978: 74.

#### Redescription.

Male: n=8. Total body length: 12.4-13.0; head length: 0.8-1.1; head width: 1.4-1.5; eye width: 0.2-0.3; interocular space: 0.8; preocellar distance: 0.3; interocellar space: 0.2. Rostrum: I 0.4-0.7, II 1.1, III 0.7, IV 0.4-0.6. Antennal segments: I 1.3-1.7, II 1.5-1.9, III 1.4-2.1 and IV 1.3. Pronotum length: 2.2-2.7; width: 3.4-3.9. Scutellum length: 1.4, wide 1.6. Length of abdomen with hemelytra: 8.0; length of abdomen with hemelytra: 9.0; abdomen width: 4.0-4.1. Dorsal coloration: Head including antennal segments 1-3 brown, 4 dark brown. Pronotum brown, margins light brown. Scutellum and corium brown tinged with dark red, hemelytral membrane dark brown. Connexival segments light brown. Ventral coloration: Ground color brown with following areas dark brown: mesosternun, metapleura, and abdomen not homogeneously dark brown. Fore- and middle legs light brown, hind leg dark brown. Structure: head granulate brown; antennal segment IV pilose; pronotum granulate. Frontal angles rugose, with a projecting as acute spines; humeral angles with two rounded projections. Scutellum granulate, dark brown. Metafemora with two rows of 6 spines; tibia with small teeth basally. Hemelytra granulated brown tinged with dark red, shorter than abdomen.

**Figures 11–14.**
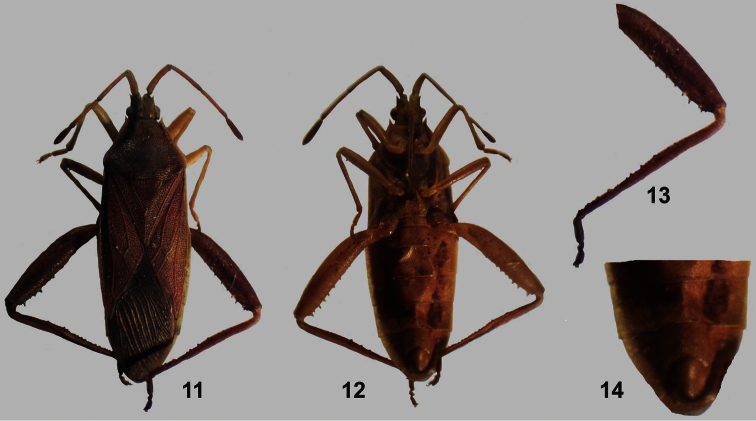
*Athaumastus subcarinatus* (Stål) ♂. **12** dorsal view **12** ventral view **13** hind leg **14** male genital capsule, ventral view.

#### Specimens examined.

**Argentina**: Buenos Aires: 1♀ Junín (34°35'16.37"S, 60°58'45.71"W). Córdoba: 1♀ Villa Nueva (32°26'08.91"S, 63°14'59.39"W). Formosa: 1♀ Almirante Brown (24°47'56.53"S, 60°27'58.83"W). Misiones: 1♀ 1♂ Loreto (27°18'59.85"S, 55°32'00.04"W).

#### Distribution.

**Argentina**: Córdoba: Carlos Paz, San Javier; Corrientes: Capital Department, Lavalle Department; Entre Ríos: Concordia Department, Federación Department, Gualeguachú Department, Paraná Department, Villaguay Department; Salta.

#### Remarks.

These are the first records of this species from Buenos Aires, Formosa, and Misiones.

### Athaumastus subterlineatus

Bergroth

http://coreoidea.speciesfile.org/Common/basic/Taxa.aspx?TaxonNameID=785

Athaumastus subterlineatus Bergroth 1912: 85

#### Distribution.

**Argentina**: Santiago del Estero, Río Salado.

### Genus Beutelspacoris

Brailovsky

http://coreoidea.speciesfile.org/Common/basic/Taxa.aspx?TaxonNameID=760

http://species-id.net/wiki/Beutelspacoris

Beutelspacoris Brailovsky 1987: 523. Type species: *Beutelspacoris sanchezi* Brailovsky 1987: 524.
Moreyacoris Casini 1989: 25. Type species: *Moreyacoris dilatata* Casini 1989: 26.

#### Diagnosis.

(After Brailovsky 1987) Body relatively small, robust, postocular tubercles prominent; antenniferous tubercles large. Pronotum slightly declivent, wider than long; callar region distinct; collar narrow; humeral angles rounded. All femora at least slightly incrassate; posterior femora more incrassate; all femora with subdistal spines on ventral surface and dorsally smooth; anterior and intermediate tibiae terete, sulcate, and unarmed; posterior tibiae slightly flattened, armed with small teeth along internal margin.

### Beutelspacoris sanchezi

Brailovsky

http://coreoidea.speciesfile.org/Common/basic/Taxa.aspx?TaxonNameID=788

Beutelpacioris sanchezi Brailovsky 1987: 524.

#### Distribution.

**Argentina**: Santiago del Estero: Lago Muyo.

### Beutelspacoris dilatata

Casini

http://coreoidea.speciesfile.org/Common/basic/Taxa.aspx?TaxonNameID=787

Moreyacoris dilatata Casini 1989: 26.–Bachmann 1999: 221.
Beutelspacoris dilatata Brailovsky and Barrera 2003: 888.

#### Distribution.

**Argentina**: Jujuy; La Rioja: Nanogasta.

### Genus Camptischium

Amyot & Serville

http://coreoidea.speciesfile.org/Common/basic/Taxa.aspx?TaxonNameID=761

http://species-id.net/wiki/Camptischium

Fig. 15

Camptischium Amyot and Serville 1843: 213. Type species: *Camptischium spinosum* Amyot and Serville 1843: 213; monotypic.

#### Diagnosis.

(After O’Shea 1980) Body-medium sized, robust, stout, elliptical or obovate; postocular tubercles relatively small, antennifers large with marked external spine; pronotum steeply declivent, humeral angles produced laterally into a sharp spine, posthumeral margins nodulose; all femora somewhat incrassate, armed with spines at least distally on ventral surface, posterior femora markedly curved, incrassate, especially in male, with tubercles ventrally and dorsally; posterior tibiae flattened, with spines situated about equidistant from anterior and lateral margins.

**Figures 15–17.**
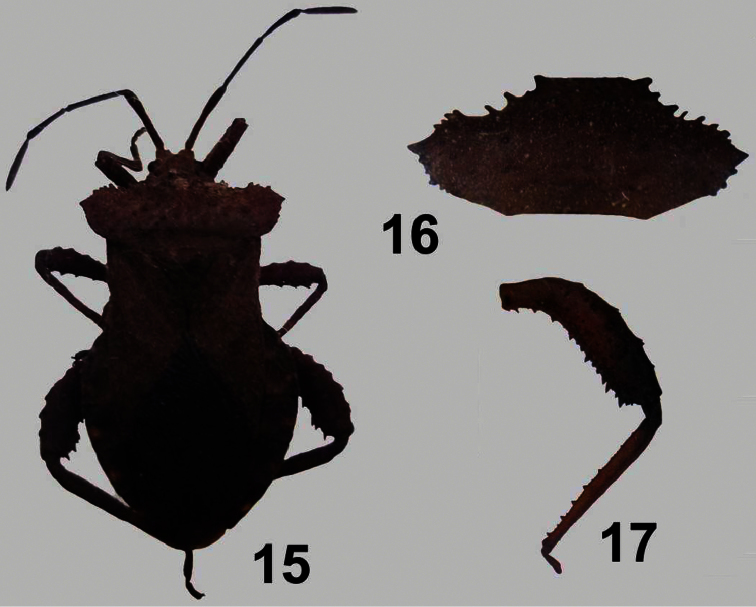
*Camptischium clavipes* (Fabricius) ♂. **15** dorsal view **16** pronotum **17** hind leg.

### Camptischium clavipes

(Fabricius)

http://coreoidea.speciesfile.org/Common/basic/Taxa.aspx?TaxonNameID=1490

http://species-id.net/wiki/Camptischium_clavipes

Figs 15–17 , 41

Coreus clavipes Fabricius 1803: 196.
Acanthocerus (Camptischium) clavipes Berg 1878: 83.–Pennington 1921: 35.
Camptischium clavipes Pennington 1920: 13.–Viana and Williner 1978: 75.
Acanthocoris clavipes Blöte 1935: 220.
Acanthocerus clavipes Bosq 1940: 401.–Hayward 1960: 30.–Quintanilla et al. 1981: 147.
Captischium clavipes Viana and Williner 1972: 27.

#### Specimens examined.

**Argentina**: Buenos Aires: 2♀ La Plata (34°55'16"S, 57°57'17"W), 1♀ Pereyra Iraola (34°50'44.08"S, 58°10'42.94"W). 1♀ 2♂ Catamarca. Córdoba: 1♀ Bella vista (29°32'07.71"S, 64°10'02.87"W), 1♂ Villa María (32°24'37.66"S, 63°14'37.12"W). 1♀ 1♂ Formosa. Jujuy: 1♀ 4♂ Yala (24°07'10.78"S, 65°24'06.78"W), 1♀ Ledesma (23°48'48.79"S, 64°47'41.47"W), 1♂ Reyes (24°09'49.08"S, 65°22'42.99"W). Salta: 2♀ 1♂ San Lorenzo (26°06'34"S, 64°38'34"W). 3♀ 4♂ Tucumán.

#### Distribution.

**Argentina**: Catamarca; Córdoba: Carlos Paz, Río San José; Chaco; Formosa; Jujuy; La Rioja; Mendoza; Misiones: Corpus, Department Cainguás, Department Montecarlo; Neuquén; Salta; San Juan; Tucumán.

#### Remarks.

This is the first record of this species from Buenos Aires.

### Genus Crinocerus

Burmeister

http://coreoidea.speciesfile.org/Common/basic/Taxa.aspx?TaxonNameID=763

http://species-id.net/wiki/Crinocerus

Fig. 18

Crinocerus Burmeister 1835: 318. Type species: *Cimex sanctus* Fabricius 1775: 709, by designation (O’Shea 1973).

#### Diagnosis.

(After O’Shea, 1980) Body medium-sized, robust, oblong; postocular tubercles well developed; antennifers large, pronounced, situated close together with distinct external spine; pronotum steeply declivent, posterior angles rounded; all femora armed at least with subdistal spines on ventral surface, posterior femora curved, incrassate especially in male, armed with tubercles on all surfaces, becoming spines on ventral surface; posterior tibiae flattened, straight in female, curved in male.

**Figures 18–20.**
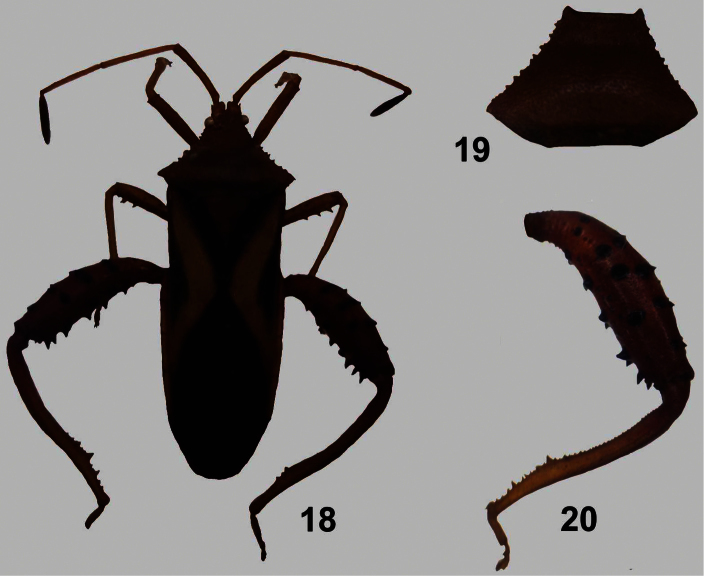
*Crinocerus sanctus* (Fabricius) ♂. **18** dorsal view **19** pronotum, 20 hind leg.

### Crinocerus sanctus

(Fabricius)

http://coreoidea.speciesfile.org/Common/basic/Taxa.aspx?TaxonNameID=792

http://species-id.net/wiki/Crinocerus_sanctus

Figs 18–20 , 42

Cimex sanctus Fabricius 1775: 709.
Crinocerus sanctus Berg 1878: 82.–Pennington 1920: 13.–Pennington 1921: 35.–Bosq 1937: 115.–Quintanilla et al. 1981: 148.

#### Specimens examined.

**Argentina**: Misiones: 2♀ 3♂ Loreto (27°18'59.85"S, 55°32'00.04"W), 3♀ 1♂ San Ignacio (27°15'34.49"S, 55°32'19.23’'W). Tucumán: 1♀.

#### Distribution.

**Argentina**: Buenos Aires; Misiones: Corpus, DepartmentAlem, Department Cainguás, Department El dorado, Department Guaraní, Department Iguazú, Department San Ignacio, Department San Javier, Santa Ana.

#### Remarks.

This is the first record of this species from Tucumán.

### Genus Dersagrena

Kirkaldy

http://coreoidea.speciesfile.org/Common/basic/Taxa.aspx?TaxonNameID=764

http://species-id.net/wiki/Dersagrena

Figs 21 –26

Dalcera Signoret 1863: 556. Type species: *Dalcera lacerdae* Signoret 1863, monotypic.
Dersagrena Kirkaldy 1904: 280. New name for *Dalcera* Signoret, takes the type for that genus.

#### Diagnosis.

(After O’Shea 1980) Body relatively small, narrow; postocular tubercles prominent, antennifers large, situated close together, pronotum relatively shallowly declivent, lateral margins slightly nodulose, humeral angles sharp, posthumeral, posterior margins smooth; anterior femora unarmed, intermediate femora armed distally on ventral surface with small spines, at least in male, posterior femora incrassate, armed with spines on ventral surface, especially in male, posterior tibiae flattened with small teeth on ventral margin, more in the male.

**Figures 21–25.**
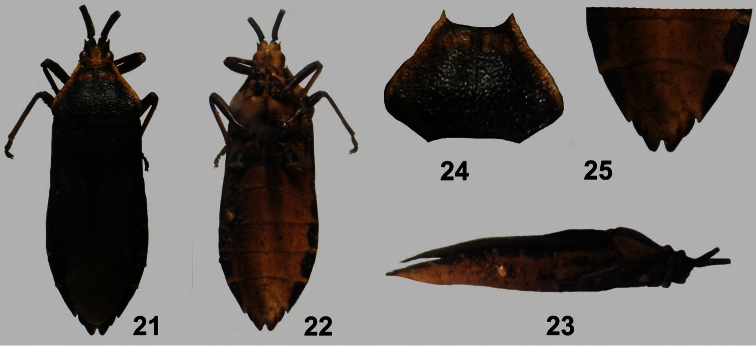
*Dersagrena flaviventris* (Berg) ♀. **21** dorsal view **22** ventral view **23** lateral view **24** pronotum **25** genital segments, ventral view.

### Dersagrena flaviventris

(Berg)

http://coreoidea.speciesfile.org/Common/basic/Taxa.aspx?TaxonNameID=793

http://species-id.net/wiki/Dersagrena_flaviventris

Figs 21–25 , 43

Dalcera flaviventris Berg 1879: 282.–Lethierry and Severin 1894: 22.
Athaumastus flaviventris Pennington 1920: 13.–Pennington 1921: 38.–Bosq 1937: 113. – Coscarón 1998: 2.
Dersagrena flaviventris Mitchell 2000: 384.

#### Redescription.

Holotype.Female. n=1. Total body length: 10.0; head length: 1.2; head width: 1.5; eye width: 0.3; interocular space: 0.7; preocellar distance: 0.2; interocellar space: 0.3. Rostrum: I 1.0, II 1.2, III 1.0, IV 0.8. Antennal segments length: I 1.1, II 1.6, 1.7 III and IV (segment missing). Pronotal length: 2.8; width: 4.1. Scutellar length: 1.4; width 1.3. Length of abdomen with male hemelytra: 9.6; length of abdomen with hemelytra: 10.0; abdomen width: 4.7. Dorsal coloration: Head dark brown, light brown interocular space. Pronotum dark brown except two stains on anterior region and lateral margins light brown. Scutellum, coria, and hemelytral membrane dark brown. Connexival segments dark brown with intersegmental line light brown. Ventral coloration: Ground color light brown, connexival segments dark brown. Legs dark brown. Structure: pronotum granulate; frontal angles granulate and projecting as acute spines reaching ocular tubercles; humeral angles with two rounded projections; scutellum granulate. Hemelytra shorter than the abdomen.

#### Specimen examined.

**Argentina**: Córdoba: 1♀ Alta Gracia (31°24'53.38"S, 64°10'36.61"W).

#### Distribution.

**Argentina**: Buenos Aires, Córdoba: Río Cuarto; Chaco; San Luis: Villa Mercedes; Santiago del Estero; Tucumán.

### Dersagrena lacerdae

(Signoret)

http://coreoidea.speciesfile.org/Common/basic/Taxa.aspx?TaxonNameID=794

Dalcera lacerdae Signoret 1863: 556.
Dersagrena lacerdae Pennington 1920: 14.–Pennington 1921: 39.

#### Distribution.

**Argentina**: Catamarca; Chaco; Formosa; Jujuy; La Rioja; Mendoza;

Misiones; Neuquén; San Juan, Tucumán.

### Dersagrena subfoveolata

(Berg)

http://coreoidea.speciesfile.org/Common/basic/Taxa.aspx?TaxonNameID=795

http://species-id.net/wiki/Dersagrena_subfoveolata

Figs 26–28 , 34–37 , 44

Athaumastus subfoveolatus Berg 1892: 66.–Bachmann 1999: 197.
Dersagrena subfoveolatus Pennington 1920: 14.–Pennington 1921: 39.
Desagrena subflaveolatus Viana and Williner 1978: 75.

#### Specimens examined.

**Argentina**: Chaco: 1♀ Corzuela (26°57'21.84"S, 60°58'14.21’'W), 1♂ Gancedo (27°29'21.89"S, 61°40'31.52"W). Formosa: 1♂ Ibarreta (25°12'55.21"S, 59°51'27.69"W). La Rioja: 1♂ Nonogasta (29°18'11.07"S, 67°30'05.67"W). Salta: 1♀ 4♂ Talapampa (25°32'00.01"S, 65°34'00.06"W). 1♂ San Luis. Santiago del Estero: 1♂ Toboada (28°02'41.53"S, 63°47'16.89"W).

#### Distribution.

**Argentina**: Catamarca; Córdoba: Capilla del Monte; Jujuy; La Pampa; La Rioja; Mendoza; Neuquén; Salta; San Juan; San Luis; Santa Fé; Santiago del Estero; Tucumán.

#### Remarks.

These are the first records of this species from Chaco and Formosa.

**Figures 26–28.**
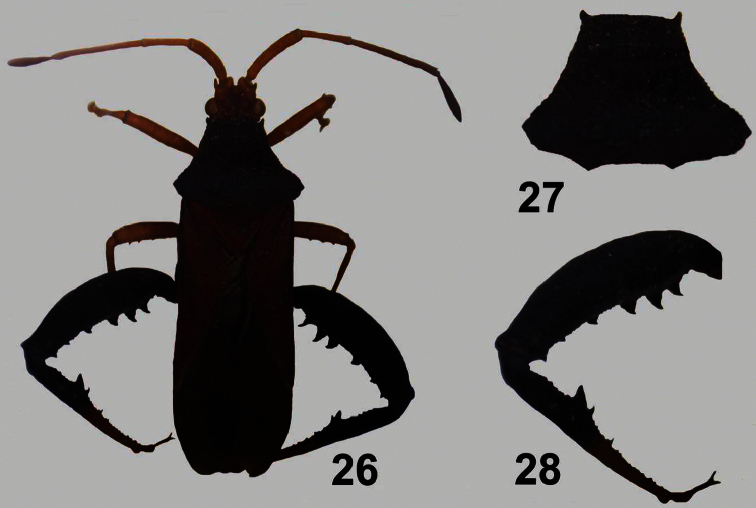
*Dersagrena subfoveolata* (Berg) ♂. **26** dorsal view **27** pronotum **28** hind leg.

### Genus Thlastocoris

Mayr

http://coreoidea.speciesfile.org/Common/basic/Taxa.aspx?TaxonNameID=774

http://species-id.net/wiki/Thlastocoris

Thlastocoris Mayr 1866: 88. Type species: *Thlastocoris laetus* Mayr, monotypic.

#### Diagnosis.

(After O’Shea 1980) Body relatively small, postocular tubercles well developed, antennifers large, fairly widely separated, external spine present on antennifers; pronotum rather shallowly declivent; all femora at least slightly incrassate, posterior femora more incrassate, a little more in male than females, anterior, intermediate femora smooth, or with obsolete spines distally on ventral surface, posterior femora with spines on ventral surface; posterior tibiae of female straight, slightly flattened, of male more flattened with widest part at midpoint, armed with teeth along ventral margin.

### Thlastocoris hernandezi

Brailovsky

http://coreoidea.speciesfile.org/Common/basic/Taxa.aspx?TaxonNameID=2210

Thlastocoris hernandezi Brailovsky 1990: 108.

#### Distribution.

**Argentina**: Formosa: Gran Guardia.

### Genus Zoreva

Amyot & Serville

http://coreoidea.speciesfile.org/Common/basic/Taxa.aspx?TaxonNameID=775

http://species-id.net/wiki/Zoreva

Fig. 29

Zoreva Amyot and Serville 1843: 216. Type species: *Coreus dentipes* Fabricius 1803: 196.

#### Diagnosis.

(After O’Shea, 1980) Body narrow and elongated. Pronotum almost hexagonal, humeral angle developed into a long sharp spine of variable length and direction, posthumeral edge with small conspicuous teeth, posterior border straight or slightly concave. Leg dimorphic. Males: Femora ventrally armed with spines of variable length, femur anterior generally smooth texture; hind femur swollen and curved. Females: Femora armed with spines ventrally, length variable anterior and middle femora thinner and a little more drawn back, but never curved.

**Figures 29–33.**
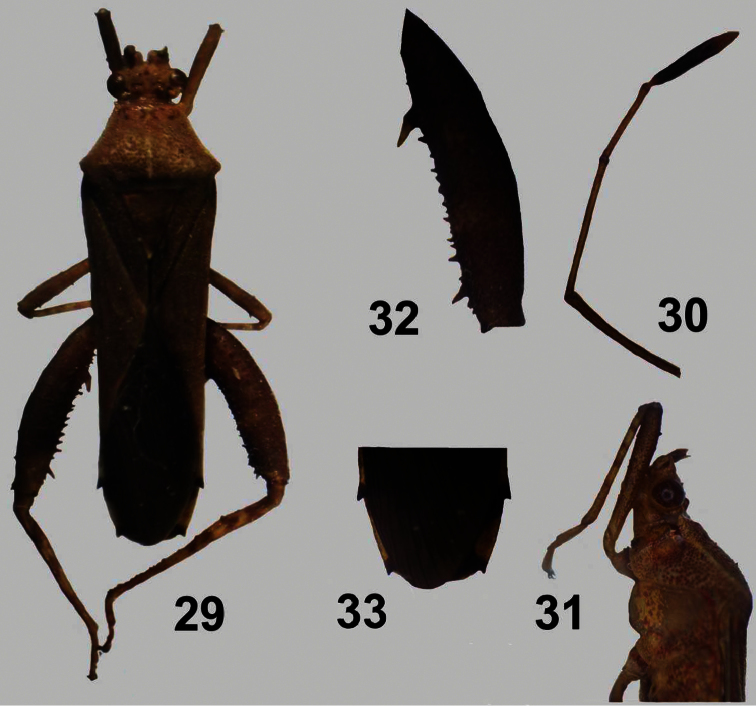
*Zoreva dentipes* Fabricius ♂. **29** dorsal view **30** antenna **31** head and pronotum **32** hind femur **33** abdomen dorsal view.

**Figures 34–37.**
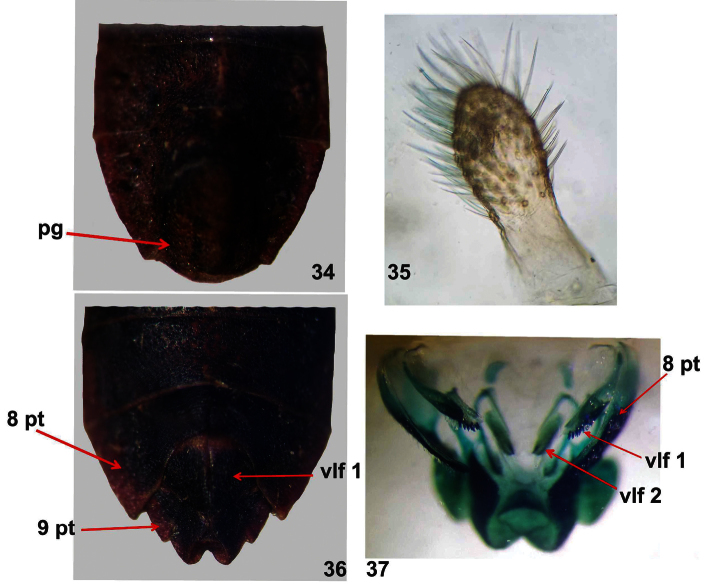
*Dersagrena subfoveolata* (Berg) ♂, ♀. **34–35** male genital capsule, ventral view **34** pg: pygophore **35** paramere **36–37** female genital segments, ventral view **36** 8 pt: paratergite eight, 9 pt: paratergite nine, vlf. 1: first valvifer **37** 8 pt: paratergite eight, vlf. 1: first valvifer, vlf. 2: second valvifer.

**Figure 38–41.**
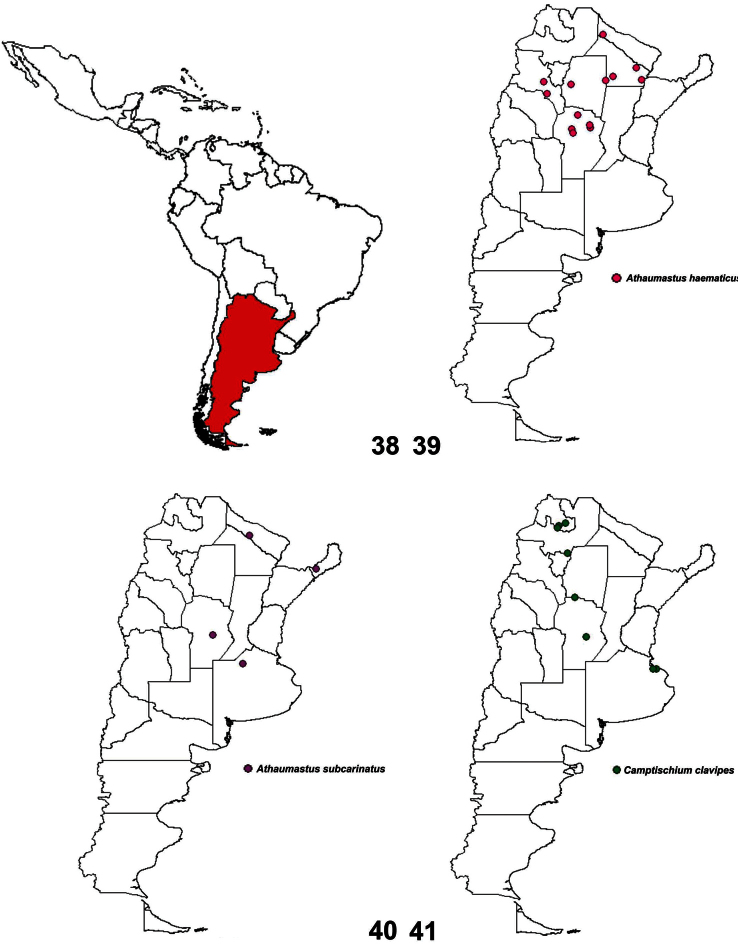
Geographical distribution: **38** Argentina geographical location **39** *Athaumastus haematicus* (Stål) **40** *Athaumastus subcarinatus* (Stål) **41** *Camptischium clavipes* (Fabricius).

**Figure 42–45.**
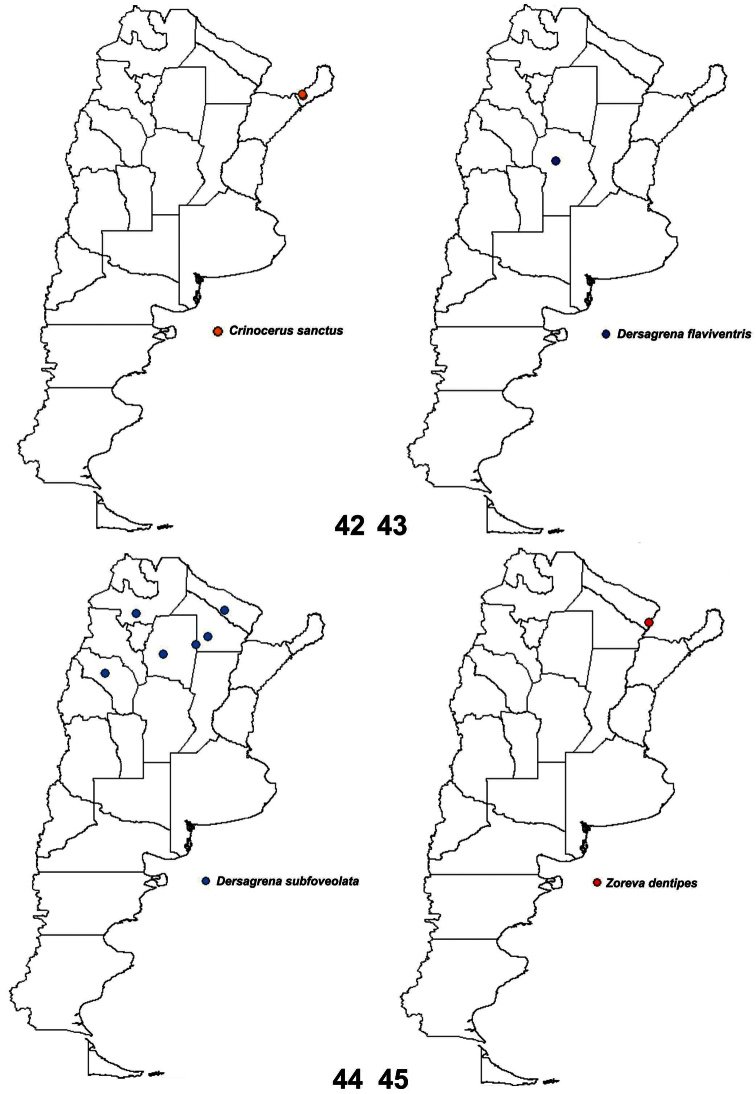
Geographical distribution: **42** *Crinocerus sanctus* (Fabricius), **43** *Dersagrena flaviventris* (Berg) **44** *Dersagrena subfoveolata* (Berg) **45** *Zoreva dentipes* Fabricius.

### Zoreva dentipes

Fabricius

http://coreoidea.speciesfile.org/Common/basic/Taxa.aspx?TaxonNameID=2516

http://species-id.net/wiki/Zoreva_dentipes

Figs 29–33 , 45

Coreus dentipes Fabricius 1803: 196.

#### Redescription.

Holotype. Male. n=1. Total body length: 9.8; head length: 1.1; head width: 1.6; eye width: 0.3; interocular space: 0.7; preocellar distance: 0.2; interocellar space: 0.3. Rostrum: (missing). Antennal segments length: I 2.6, II 2.4, III 1.4, IV 1.8. Pronotal length: 1.8; width: 2.8. Scutellar length: 1.3; width 1.1. Length of abdomen with hemelytra: 8.5; length of abdomen with hemelytra: 8.4; abdomen width: 2.4. Dorsal coloration: Head light brown, except anterior region of ocelli and post ocular region dark brown, with many short hairs. Antennal segments 1-2 longer than 3-4, 4 longer than 3, segment 1-3 light brown, segment 4 dark brown except base, and segment 5 light brown. Pronotum are light brown except anterior margin dark brown. Scutellum brown except edges and posterior process dark brown. Connexival: two lateral segment light brown except lateral projection dark brown. Ventral coloration: light brown except area next to conexivum dark brown to brown, light brown not uniformy red. Legs: fore and middle femora dark brown, tarsi light brown with light brown spots. Mid femur with one spine distally. Fore femur dark brown with spines, basally very long, medially short and distally long. Structure: pronotum granulose; frontal angles granulose and projecting as acute spines reaching ocular tubercles; humeral angles with two rounded projections and shorter spines; scutellum granulose with pilosity. Hemelytra as long as the abdomen, brown with punctuations and pilosity, membrane dark brown.

#### Specimen examined.

**Argentina**: Formosa: 1♂ Laguna Oca (26°13'56.25"S, 58°13'04.84"W).

#### Distribution.

**Argentina**: Formosa: Laguna Oca.

#### Remarks.

This is the first record of this species for Argentina

## Supplementary Material

XML Treatment for Athaumastus

XML Treatment for Athaumastus haematicus

XML Treatment for Athaumastus macer

XML Treatment for Athaumastus subcarinatus

XML Treatment for Athaumastus subterlineatus

XML Treatment for Beutelspacoris

XML Treatment for Beutelspacoris sanchezi

XML Treatment for Beutelspacoris dilatata

XML Treatment for Camptischium

XML Treatment for Camptischium clavipes

XML Treatment for Crinocerus

XML Treatment for Crinocerus sanctus

XML Treatment for Dersagrena

XML Treatment for Dersagrena flaviventris

XML Treatment for Dersagrena lacerdae

XML Treatment for Dersagrena subfoveolata

XML Treatment for Thlastocoris

XML Treatment for Thlastocoris hernandezi

XML Treatment for Zoreva

XML Treatment for Zoreva dentipes
